# Heteroresistance to Amikacin in Carbapenem-Resistant *Klebsiella pneumoniae* Strains

**DOI:** 10.3389/fmicb.2021.682239

**Published:** 2021-12-02

**Authors:** Feiyang Zhang, Qin Li, Jiawei Bai, Manlin Ding, Xiangjin Yan, Guangxi Wang, Baoli Zhu, Yingshun Zhou

**Affiliations:** ^1^Department of Pathogen Biology, School of Basic Medicine, Public Center of Experimental Technology of Pathogen Biology Technology Platform, Southwest Medical University, Luzhou, China; ^2^Key Laboratory of Pathogenic Microbiology and Immunology, Institute of Microbiology, Chinese Academy of Sciences, Beijing, China

**Keywords:** *Klebsiella pneumoniae*, amikacin, heteroresistance, carbapenem, multidrug resistance

## Abstract

Heteroresistance can lead to treatment failure and is difficult to detect by the methods currently employed by clinical laboratories. The aim of this study was to investigate the prevalence of the amikacin-heteroresistant *Klebsiella pneumoniae* strains and explore potential amikacin heteroresistance mechanism through whole-genome sequencing (WGS) and quantitative reverse-transcription PCR (qRT-PCR). In this study, 13 isolates (8.39%) were considered as amikacin-heteroresistant *K. pneumoniae* strains among a total of 155 *K. pneumoniae* strains. The majority of the heterogeneous phenotypes (11/13, 84.61%) was unstable and the minimal inhibitory concentrations (MICs) fully or partially reverted back to the level of susceptibility of the parental isolate. The frequency of heteroresistant subpopulation ranged from 2.94×10^−7^ to 5.59×10^−6^. Whole-genome sequencing and single-nucleotide variants (SNVs) analysis showed that there were different nucleotide and resultant amino acid alterations among an amikacin-heteroresistant strain S38 and the resistant subpopulation S38L in several genes. Quantitative reverse-transcription PCR analysis revealed that the increased expression of aminoglycoside resistance genes detected in amikacin-heteroresistant *K. pneumoniae* strains might be associated with amikacin heteroresistance. The findings raise concerns for the emergence of amikacin-heteroresistant *K. pneumoniae* strains and the use of amikacin as therapy for the treatment of multidrug-resistant *K. pneumoniae* strains.

## Introduction

Heteroresistance refers to a phenomenon where there are different subpopulations of seemingly isogenic bacteria which exhibit a range of susceptibilities to antibiotics (El-Halfawy and Valvano, 2015). Heteroresistance has been reported to a wide variety of antibiotics and is very common in several bacterial species (Anderson et al., 2018). The mechanism of heteroresistance is very complex. As previously reported, the high prevalence of antibiotic heteroresistance was mainly caused by spontaneous tandem genes amplification, typically including known resistance genes (Nicoloff et al., 2019). In addition, point mutations, insertion sequences (IS), insertions or small deletions, overexpression of the antibiotic resistance gene, reduced expression of the porin-encoding gene, biofilm formation, and slow growth were also linked to heteroresistance (Adams-Sapper et al., 2015; Silva et al., 2016; Gallagher et al., 2017; He et al., 2018; Rodriguez et al., 2019; Jia et al., 2020). The phenomenon of heteroresistance in *Klebsiella pneumoniae* has been reported to a variety of antibiotics, including β-lactams, colistin, and tetracyclines (Tato et al., 2010; Jayol et al., 2015; Zheng et al., 2018). However, heteroresistance to aminoglycoside was extremely rare (Anderson et al., 2018). In 1947, heteroresistance was first reported in which researchers found that *Haemophilus influenzae* contained rare subpopulation with increased streptomycin resistance (El-Halfawy and Valvano, 2015). In 2014, researchers found that decreased expression of the porin gene *ompC* was associated with the presence of kanamycin-resistant subpopulation in *Salmonella enterica* (Sánchez-Romero and Casadesús, 2014). Recently, researchers found that heteroresistance to tobramycin in *Acinetobacter baumannii* was related to the amplification of aminoglycoside resistance gene *aadBc* (Anderson et al., 2018). However, the prevalence and mechanism of amikacin heteroresistance in *K. pneumoniae* are still not clear. In a previous study at our center about the susceptibility of *K. pneumoniae* clinical isolates to different antibiotics using the disk diffusion method, the appearance of distinct colonies growing inside the clear zone of inhibition in amikacin disk was found. This phenomenon has attracted our attention and was defined as heteroresistance by relevant articles. As previously reported, heteroresistance could be divided into three forms (Li et al., 2017). In the first form, the entire population was susceptible to the antibiotic, whereas the MICs of subpopulations were distinct. In the second form, the population was susceptible to an antibiotic, with a few highly resistant subpopulations. Finally, in the third form, the entire population was resistant or intermediate resistant with a group of subpopulations showing increased resistance to the antibiotic. The reason for selecting S38 for whole-genome sequencing (WGS) and single-nucleotide variants (SNVs) analysis was that S38 is the third form of heteroresistance. The major concern about this form was that more resistant subpopulation may pass a switch of heteroresistance to homogeneous high-level resistance and could pose a serious threat to antibiotic treatment. In addition, this type of heteroresistance could help us understand that heteroresistance was considered as an intermediate stage of resistance. Beyond that, biofilm formation, growth rate analysis, and quantitative reverse-transcription PCR (qRT-PCR) were also performed to explore the potential amikacin heteroresistance mechanism in the current study.

In brief, in this study, we investigated the prevalence of the amikacin-heteroresistant *K. pneumoniae* strains and explored the potential amikacin heteroresistance mechanism. The findings could help us understand the switch of heteroresistance to homogeneous high-level resistance, raise our concerns for the use of amikacin as therapy for the treatment of multidrug-resistant *K. pneumoniae*, and direct the appropriate use of antibiotics in clinic.

## Materials and Methods

### Bacterial Strains, Antimicrobial Susceptibility Testing, and Resistance Genes Detection

This study of nosocomial *K. pneumoniae* infections was performed from January 2019 to December 2019 in six hospitals in Sichuan, Henan, Hunan, and Fujian provinces and the city of Shanghai in China. A total of 155 non-duplicate *K. pneumoniae* isolates recovered from sterile body fluids, sputum, and wound secretion were included in this study. We chose the first isolate from the patients and we also excluded patients with polymicrobial infections or concurrent sterile body fluid infection with an organism other than *K. pneumoniae*. Sterile body fluids were defined as specimens that were collected from sterile sites with sterile procedures, such as blood and cerebrospinal fluid. Routine species identification was carried out by the Vitek2 system (BioMérieux, Marcy-l’Étoile, France). Susceptibility to different antibiotics (imipenem, meropenem, amikacin, cefoperazone, and ciprofloxacin) was determined by the method of disk diffusion according to the guidelines of the Clinical and Laboratory Standards Institute (CLSI; CLSI, 2019). Heteroresistance to different antibiotics was screened by the method of disk diffusion and the appearance of distinct colonies growing inside the clear zone of inhibition was considered as heteroresistance (He et al., 2018). Briefly, the heteroresistant strains were isolated on LB agar plates, a single colony was selected, and its turbidity was adjusted to a concentration of 0.5 McFarland with sterile saline. The bacterial suspensions were evenly plated on the MH plates and dried at room temperature for 3min. The antibiotic disk was affixed on the MH plates with sterile forceps. After incubation at 35°C for 18h, the diameter of the inhibition zone was measured. The minimal inhibitory concentrations (MICs) were determined by microbroth dilution method according to the guidelines of the CLSI with seven antibiotics: amikacin, imipenem, meropenem, ciprofloxacin, kanamycin, chloramphenicol, and cefotaxime. *Escherichia coli* ATCC25922 was used as the quality control strain. The resistance breakpoints for antimicrobial agents were determined according to the recommendations of the CLSI (CLSI-M100-S29; CLSI, 2019). PCR amplification and DNA sequencing were performed to identify the key carbapenemase encoding genes (*bla*_NDM_, *bla*_KPC_, *bla*_VIM_, *bla*_IMP_, and *bla*_OXA-48_), the key extended-spectrum beta-lactamase (ESBLs) genes (*bla*_CTX-M_, *bla*_SHV_, and *bla*_TEM_), the common aminoglycoside-modifying enzymes (AMEs) genes [*aac (3)-I*, *aac (3)-II*, *aac (6′)-I*, *aac (6′)-II*, *ant (2″)-I*, *ant (3″)-I*, *aph (3′)-I*, and *aph (4)-Ia*], and common 16sRNA methylase genes (*armA*, *rmtA*, *rmtB*, *rmtC*, and *rmtD*) as previously described (Yin et al., 2008; Zhou et al., 2010; Poirel et al., 2011; Miró et al., 2013; Fernández-Martínez et al., 2015).

### Population Analysis Profile

Heteroresistance to amikacin in *K. pneumoniae* strains was confirmed by PAP. PAP was performed by the method described previously (Lin et al., 2020). *Klebsiella pneumoniae* strains were inoculated in LB broth. After overnight incubation at 37°C, the cultures were diluted with sterile saline to approximately 10^8^CFU/ml (CFU, Colony-Forming Unit). Then, 100μl of the cultures was taken and plated on LB agar plates containing increasing concentrations of amikacin (0, 8, 16, 32, 64, and 128μg/ml). Plates were then incubated at 37°C, and CFU was enumerated after 48h. *Klebsiella pneumoniae* ATCC13883 was used as the control strain. The resistant subpopulation of each amikacin-heteroresistant isolate was selected from the 64μg/ml amikacin concentration of the PAP test. The frequency of heteroresistant subpopulation was calculated by dividing the number of colonies grown on plate containing the highest antibiotic concentration by the number of colonies grown on antibiotic-free plate. The experiments were repeated three times.

### Stability of the Heterogeneous Phenotype

Stability of the heterogeneous phenotype was performed by the method described previously (Nicoloff et al., 2019). Briefly, two to four clones with decreased susceptibility were selected from PAP test plates containing amikacin at a concentration of 64μg/ml (the highest concentration at which the amikacin-heteroresistant *K. pneumoniae* strains could grow), re-isolated on LB plate and grown overnight in LB broth. The MICs of these clones were determined by the microbroth dilution method. The cultures were then grown for 7days in absence of amikacin and the daily subcultures were performed (1μl of the cultures inoculated into 1ml of LB broth, 10 generations per day). The MICs after 7days in absence of selective pressure were determined and the heterogeneous phenotype was deemed unstable if the MICs decreased or reverted to that of the original parental isolate in at least one of the cultures. The experiments for determining the stability of the heterogeneous phenotype were repeated three times.

### *In-vitro* Induction of Amikacin Resistance in *K. pneumoniae* Strains Under Amikacin Pressure

*In-vitro* induction of amikacin resistance in *K. pneumoniae* strains was performed by the method described by Lin et al. (2020). Thirteen amikacin-heteroresistant isolates were used to induce amikacin resistance isolates. Briefly, these isolates were inoculated in LB broth. After overnight incubation at 37°C, the cultures were diluted with sterile saline to approximately 10^8^CFU/ml. Then, 100μl of the cultures was taken and sub-cultured serially in 1ml LB broth containing gradually increasing concentrations of amikacin with the initial concentration being 2× MIC values followed by successive increases to 4× and 8× MICs. *K. pneumoniae* strains were cultured for four passages before their entry into the next concentration. Isolates from the passages of each concentration were stored at −80°C in LB broth containing 50% glycerol. Then, the MICs and the proportion of resistant subpopulation of these isolates were determined. The proportion of resistant subpopulation was determined by dividing the number of colonies grown on plate containing amikacin at a concentration of 64μg/ml by the number of colonies grown on antibiotic-free plate. Then, the isolates were sub-cultured for 7days in absence of amikacin (1μl of the cultures inoculated into 1ml of LB broth, 10 generations per day). The MICs and the proportion of resistant subpopulation were determined again.

### MLST Analysis

MLST analysis was performed as previously described by Pasteur Institute MLST Database. PCR amplification for seven housekeeping genes (*gapA*, *infB*, *mdh*, *pgi*, *phoE*, *rpoB*, and *tonB*) was carried out using primers and protocols available at the Pasteur Institute MLST Database^1^ (Nava et al., 2019). The allelic profiles and sequence types (ST) were available in the MLST database^2^ (Huang et al., 2018).

### Measurement of Bacterial Growth Curves

Growth curves of amikacin-resistant parental strain and their resistant subpopulation were determined as previously described (Jayol et al., 2015). Briefly, *K. pneumoniae* strains were inoculated into 5ml of LB broth and incubated overnight at 37°C. Overnight cultures were diluted to approximately 10^8^CFU/ml and inoculated in 2ml of LB broth. The cultures were grown at 37°C and 200rpm for 12h. The absorbance at the optical density of 570nm was determined every hour for 12hours; then, the growth curves were plotted.

### Biofilm Formation

Biofilm formation was determined as previously described (Vuotto et al., 2017). Amikacin-resistant parental strains and resistant subpopulation were inoculated in LB broth at 37°C and 200rpm for 16h. The concentration of the cultures was adjusted to approximately 10^8^CFU/ml; 200μl of the cultures was added into the 96-well plate and incubated at 37°C for 48h. Each well was washed with 200μl phosphate-buffered saline (PBS) three times to remove planktonic bacteria. After staining with 0.1% crystal violet for 30min, the crystal violet was washed thoroughly with PBS and the 96-well plate was dried at room temperature. Then, 200μl of 95% ethanol was added into each well to dissolve crystal violet. The absorbance of dissolved dye was measured at 570nm (OD_570_). *Klebsiella pneumoniae* strain NTUH-K2044 was selected as the positive control and LB broth as the negative control. The OD cutoff (ODc) was defined as three SDs above the mean OD of the negative control. All the strains were classified into the following categories: non-biofilm producers (OD≤ODc), weak biofilm producers (ODc<OD≤2× ODc), moderate biofilm producers (2× ODc<OD≤4× ODc), and strong biofilm producers (4× ODc<OD). Each assay was performed in triplicate and repeated four times.

### Quantitative Reverse-Transcription PCR Analysis

The expression levels of aminoglycoside resistance genes *aac (6′)-I*, *aph (3′)-Ia*, *aac (3)-II*, and porin genes *ompK35* and *ompK36* were assessed using qRT-PCR. The primers used in qRT-PCR analysis can be obtained in the supplementary material in Supplementary Table S1. Amikacin-heteroresistant *K. pneumoniae* strains and their resistant subpopulation were grown to midlogarithmic phase and total RNA was extracted by using Trizol reagent (Invitrogen, Carlsbad, CA, United States). Then, the extracted RNA was reverse transcribed into cDNA using a PrimeScript RT reagent kit (Takara Bio Inc., Shiga, Japan). Finally, qRT-PCR was performed using a SYBR Premix Ex Taq II kit (Takara Bio Inc., Shiga, Japan) in a Mastercycler ep realplex system (Eppendorf, Hamburg, Germany), with an initial incubation at 94°C for 30s, followed by 40cycles of 5s at 94°C and 30s at 60°C. The internal control gene 16S rRNA was used to normalize the expression of each candidate gene. The threshold cycle (Ct) numbers were determined by the detection system software, and the data were analyzed based on the 2^−ΔΔCt^ method. The experiments were carried out in triplicate with three independent RNA samples.

### Whole-Genome Sequencing and Bioinformatics Analysis

The genomic DNA was extracted using a DNA extraction kit (TIANGEN, Beijing, Co Ltd.). The genome was sequenced by the Illumina PE300 platforms (Majorbio Co., Ltd. Shanghai, China).

The clean reads were assembled using the SOAPdenovo software package (Luo et al., 2012). GeneMarkS software^3^ was used to make predictions about the encoding genes (Li et al., 2017). RASTv.2.0^4^ was used to complete bacterial genome annotations. The predicted genes were compared to a nonredundant (nr) protein database in NCBI using BLASTX. rRNA, tRNA, and sRNAs were predicted by rRNAmmer, tRNAscan software, and Rfam database, respectively (Lagesen et al., 2007; Nawrocki et al., 2015; Lowe and Chan, 2016). Repetitive sequences were predicted using RepeatMasker^5^ (Saha et al., 2008). Functional classification was performed by aligning the predicted proteins to the Clusters of Orthologous Groups (COG) database (Tatusov et al., 2003). Metabolic pathways were analyzed by a Diamond software on the KEGG web server.^6^ Diamond software was used to compare the amino acid sequence with the Virulence Factors of Pathogenic Bacteria database. Antimicrobial resistance genes were identified using the ResFinder 4.1 tool^7^ (Champion et al., 2009). The genomic islands (GIs) were predicted by using the IslandPath-DIOMB software (Hsiao et al., 2003). PhiSpy software was used to predict Prophage (Akhter et al., 2012). CRISPR digger was used for CRISPR identification (Ge et al., 2016). Single-nucleotide variants analysis was determined by whole-genome alignment and using the MegAlign software package.

The genome sequences of the S38 and S38L were submitted to the GenBank under accession numbers JAGFBW000000000 and JAGFBX000000000.

### Statistical Analysis

Student’s *t* test with GraphPad Prism software 7.0 was used to analyze the continuous data obtained by biofilm formation analysis and qRT-PCR analysis. The differences were considered statistically significant when value of *p*<0.05.

## Results

### Antibiotic Susceptibility Results of *K. pneumoniae*

Antibiotic susceptibility results for different antibiotics in 155 strains of *K. pneumoniae* are listed in Table 1. The general information of the 155 *K. pneumoniae* isolates is listed in Supplementary Table S2 in the supplementary material. As shown in Table 1, 18 (11.61%) strains of *K. pneumoniae* were resistant to imipenem, 55 (35.48%) strains were resistant to meropenem, 59 strains (38.06%) were resistant to amikacin, 103 (66.45%) strains were resistant to cefoperazone, and 97 (62.58%) strains were resistant to ciprofloxacin. Besides, the prevalence of carbapenem heteroresistance was high in all tested strains. A total of 38 isolates (24.52%) were identified as heteroresistant to meropenem and a high prevalence (48.39%) of imipenem heteroresistance was also observed. Besides, 24 isolates of *K. pneumoniae* (15.48%) were identified as heteroresistant to cefoperazone, 13 isolates (8.39%) were identified as heteroresistant to amikacin.

**Table 1:** The distribution of antibiotic susceptibility results for different antibiotics in 155 strains of *K. pneumoniae* (*n*, %).

Antibiotics	Antimicrobial susceptibility			
	S^a^	I^b^	R^c^	HR^d^
Imipenem	54 (34.84%)	8 (5.16%)	18 (11.61%)	75 (48.39%)
Meropenem	58 (37.42%)	4 (2.58%)	55 (35.48%)	38 (24.52%)
Amikacin	48 (30.97%)	35 (22.58%)	59 (38.06%)	13 (8.39%)
Cefoperazone	19 (12.26%)	9 (5.81%)	103 (66.45%)	24 (15.48%)
Ciprofloxacin	41 (26.45%)	17 (10.97%)	97 (62.58%)	0 (0%)

a *S: K. pneumoniae strains were susceptible to antibiotics*.

b *I: K. pneumoniae strains were intermediately resistant to antibiotics*.

c *R: K. pneumoniae strains were resistant to antibiotics*.

d *HR: K. pneumoniae strains were heteroresistant to antibiotics*.

### Antimicrobial Susceptibility Profiles of the Amikacin-Heteroresistant *K. pneumoniae*

Antibiotic susceptibility results, the resistance phenotypes, the distribution of carbapenenmase, ESBLs encoding genes, aminoglycoside-modifying enzymes encoding genes (AMEs), and 16sRNA methylase genes are listed in Table 2. These strains were resistant to more than two different types of antibiotics and the majority of the amikacin-heteroresistant strains could be classified as multidrug-resistant (MDR) *K. pneumoniae*. It was observed that eight strains (61.54%) of amikacin-heteroresistant *K. pneumoniae* were resistant to kanamycin, 11 strains (84.6%) were resistant to ciprofloxacin, and eight strains (61.54%) were resistant to chloramphenicol. All the identified strains were resistant to cefotaxime and at least one carbapenems. In other words, the amikacin-heteroresistant strains belonged to carbapenem-resistant *K. pneumoniae* (CRKP) strains and the vast majority of the heteroresistant *K. pneumoniae* strains carried at least two β-lactam resistance genes. Aminoglycoside resistance genes *aac (6′)-Ib*, *aph (3′)-Ia* were detected in eight isolates (61.54%) and *aac (3)-II* was detected in 11 isolates (84.6%). 16sRNA methylase genes were not detected in these amikacin-heteroresistant *K. pneumoniae* strains.

**Table 2:** Resistance phenotypes, distribution of carbapenenmase, ESBLs, AMEs encoding genes and 16sRNA methylase genes, sequence typing, and biofilm formation ability in amikacin-heteroresistant *K. pneumoniae* strains.

Strains	MIC (μg/ml)							Carbapenenmase, ESBLs, 16sRNA methylase genes and AMEs encoding genes	Sequence type	Biofilm formation ability
	AMK	IMP	MEM	CIP	KAN	CHL	CTX			
S38	32 (I^a^)	64 (R^b^)	128 (R)	128 (R)	2048 (R)	1,024 (R)	128 (R)	*bla*_KPC-2_, *bla*_OXA-1_, *bla*_SHV-182_, *bla*_TEM-1B_, *bla*_CTX-M-15_, *aac (6')-Ib-cr*, *aph (3’)-Ia*	ST11	Moderate
S33^*^	4 (S^c^)	64 (R)	256 (R)	16 (R)	128 (R)	4 (S)	64 (R)	*bla*_CTX-M-65_, *bla*_TEM-1B_, *bla*_SHV-12_, *aac (3)-IId*, *aac (6')-Ib-cr*	ST2324	Weak
S30^*^	4 (S)	4 (R)	2 (I)	8 (R)	64 (R)	32 (R)	64 (R)	*bla*_CTX-M-3_, *bla*_TEM-1B_, *bla*_SHV-27_, *bla*_VIM-2_, *aac (6')-Ib3*, *aac (3)-IId*	ST11	Weak
S28^*^	4 (S)	4 (R)	2 (I)	4 (R)	128 (R)	32 (R)	64 (R)	*bla*_CTX-M-15_, *bla*_TEM-1B_, *bla*_SHV-11_, *bla*_VIM-4_, *aac (3)-IIa*, *aac (6')-Ib3*	ST307	Moderate
S9	2 (S)	32 (R)	16 (R)	32 (R)	2048 (R)	64 (R)	64 (R)	*bla*_KPC-2_, *bla*_NDM-5_, *bla*_CTX-M-27_, *bla*_TEM-1B_, *bla*_IMP-4_, *bla*_SHV-18_, *aac (3)-IIa*, *aac (6')-Ib-cr*	ST11	Moderate
F48	2 (S)	32 (R)	64 (R)	64 (R)	8 (S)	128 (R)	64 (R)	*bla*_KPC-2_, *bla*_CTX-M-15_, *bla*_TEM-116_, *bla*_SHV-29_, *bla*_VIM-1_, *aph (3')-Ia*, *aac (3)-IIa*	ST11	Weak
F50	2 (S)	16 (R)	2 (I)	0.25 (S)	8 (S)	4 (S)	32 (R)	*bla*_NDM-5_, *bla*_CTX-M-1_, *bla*_TEM-1B_, *bla*_SHV-182_, *aph (3')-Ia*	ST11	Moderate
F35	8 (S)	16 (R)	8 (R)	8 (R)	256 (R)	32 (R)	128 (R)	*bla*_CTX-M-3_, *bla*_TEM-1B_, *bla*_SHV-33_, *bla*_IMP-4_, *aph (3')-Ia*, *aac (3)-IId*, *aac (6')-Ib-cr*	ST2637	Moderate
H28	4 (S)	64 (R)	4 (R)	0.25 (S)	8 (S)	4 (S)	64 (R)	*bla*_CTX-M-15_, *bla*_TEM-1B_, *bla*_SHV-144_, *bla*_IMP-2_, *aph (3')-Ia*, *aac (3)-IIa*	ST2637	Weak
H628^*^	16 (S)	64 (R)	8 (R)	128 (R)	2048 (R)	256 (R)	64 (R)	*bla*_CTX-M-65_, *bla*_TEM-1B_, *bla*_SHV-27_, *bla*_VIM-4_, *aph (3')-Ia*, *aac (3)-IIa*, *aac (6')-Ib-cr*	ST11	Weak
17-3	2 (S)	64 (R)	128 (R)	32 (R)	16 (S)	4 (S)	32 (R)	*bla*_KPC-2_, *bla*_TEM-1B_, *bla*_SHV-55_, *bla*_VIM-4_, *bla*_IMP-4_, *aac (3)-IIa*, *aph (3')-Ia*	ST11	Moderate
17-4	8 (S)	64 (R)	128 (R)	16 (R)	8 (S)	128 (R)	64 (R)	*bla*_KPC-2_, *bla*_NDM-5_, *bla*_CTX-M-1_, *bla*_TEM-1B_, *bla*_SHV-2_, *bla*_VIM-2_, *aac (3)-IId*, *aph (3')-Ia*	ST11	Moderate
17-10^*^	8 (S)	64 (R)	2 (I)	128 (R)	64 (R)	16 (I)	64 (R)	*bla*_CTX-M-1_, *bla*_TEM-1B_, *bla*_SHV-48_, *aac (3)-IIa*, *aac (6')-Ib-cr*	ST761	Moderate

*IMP, imipenem; MEM, meropenem; CTX, cefotaxime; CIP, ciprofloxacin; CHL, chloramphenicol; and KAN, kanamycin*.

a *I: K. pneumoniae strains were intermediately resistant to antibiotics*.

b *R: K. pneumoniae strains were resistant to antibiotics*.

c *S: K. pneumoniae strains were susceptible to antibiotics*.

* *K. pneumoniae isolates which do not have carbapenemases but are resistant to imipenem or meropenem. It is speculated that the reduced susceptibility to imipenem or meropenem might be due to porins, which is already established*.

### Characterization of the Amikacin-Heteroresistant *K. pneumoniae*

**Figure 1:**
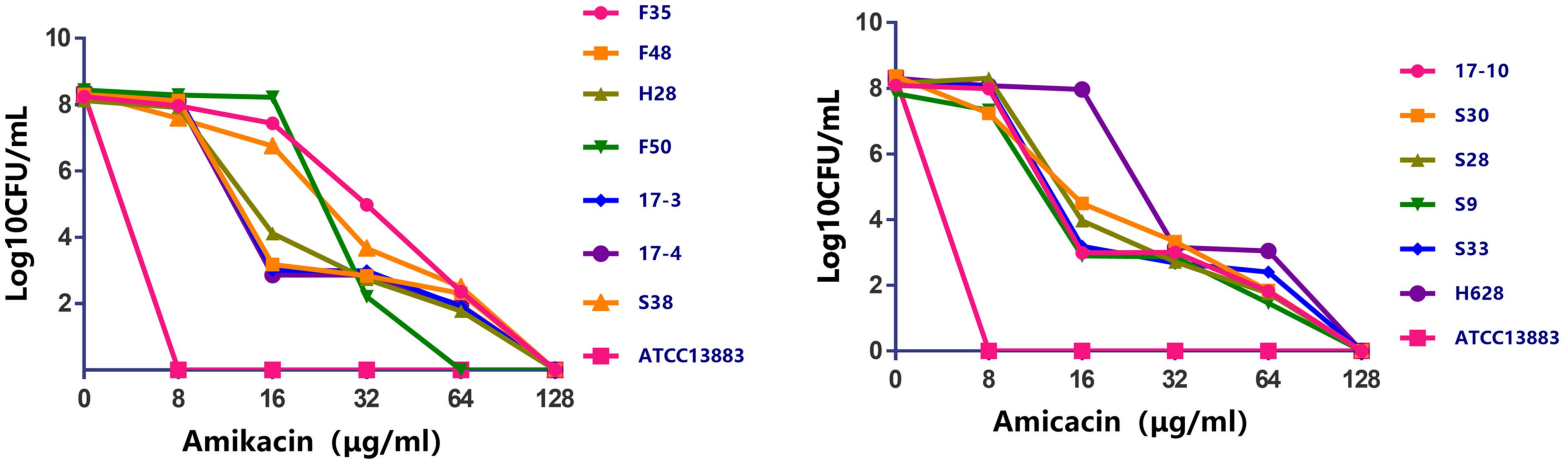
Population analysis profiles of the amikacin-heteroresistant *Klebsiella pneumoniae* isolates and the control strain *K. pneumoniae* ATCC13883. The *x* axis indicates the amikacin concentration in micrograms per milliliter used to select amikacin-resistant subpopulation with higher amikacin resistance levels, and on the *y* axis, the frequency of bacterial cells is given as the logarithm to the base 10 of CFU per milliliter. Dots correspond to mean values of three replicates for each strain.

Characteristics of amikacin-heteroresistant *K. pneumoniae* strains are shown in Table 3. The MICs of amikacin for the *K. pneumoniae* clinical isolates ranged from 2 to 32μg/ml; however, the heteroresistant subpopulation grown within the zone of inhibition around amikacin disks showed MICs of 64μg/ml or 128μg/ml. PAP showed that subpopulation of the amikacin-heteroresistant *K. pneumoniae* strains could grow at concentrations ranging from 8 to 64μg/ml (4–32 times their MICs), while the control isolate (*K. pneumoniae* ATCC13883) could not grow at a concentration of 2μg/ml (Figure 1). The vast majority of the heterogeneous phenotypes was unstable except *K. pneumoniae* strains S38 and F35 which were stable after seven daily subcultures in antibiotic-free medium.

**Table 3:** Characteristics of the amikacin-heteroresistant *K. pneumoniae* strains.

Isolates	Amikacin MIC (μg/ml)	Highest amikacin concentration for growth in PAP test (μg/ml)	MIC of heterogeneous subpopulation (μg/ml)	MIC of heterogeneous subpopulation grown for 7days without amikacin (μg/ml)	PAP frequency	Heterogeneous phenotype
S38	32	64	128	128	2.22×10^−6^	Stable
S33	4	64	64	8	1.68×10^−6^	Unstable
S30	4	64	64	16	2.94×10^−7^	Unstable
S28	4	64	128	8	4.11×10^−7^	Unstable
S9	2	64	64	2	4.20×10^−7^	Unstable
F48	2	64	64	4	1.01×10^−6^	Unstable
F50	2	32	32	8	5.98×10^−7^	Unstable
F35	8	64	64	64	1.33×10^−6^	Stable
H28	4	64	128	8	4.50×10^−7^	Unstable
H628	16	64	128	4	5.59×10^−6^	Unstable
17-3	2	64	64	2	4.78×10^−7^	Unstable
17-4	8	64	64	8	3.45×10^−7^	Unstable
17-10	8	64	64	8	5.49×10^−7^	Unstable
ATCC13883	<2	ND^a^	ND	ND	ND	ND

*MIC, minimal inhibitory concentration and PAP, population analysis profile*.

a *ND means these experiments were not performed*.

### Results of Induction of Amikacin Resistance in *K. pneumoniae* Under Amikacin Pressure

The results of inducing amikacin resistance in *K. pneumoniae* strains under amikacin pressure are shown in Table 4. The MICs of strains under amikacin pressure were 4- to 32-fold higher than their initial MICs. When isolates were sub-cultured serially in LB broth containing gradually increasing concentrations of amikacin, the proportion of resistant subpopulation also increased along with increasing amikacin concentration. All the tested strains evolved to intermediate or complete resistance to amikacin under amikacin pressure. The MICs and the proportion of resistant subpopulation were decreased to different extent in the strains whose heterogeneous phenotypes were unstable after they were sub-cultured for 7days without amikacin. However, different results were found in the strains whose heterogeneous phenotypes were stable. The MICs and the proportion of resistant subpopulation in these strains could be maintained.

**Table 4:** Results of inducing amikacin resistance in *K. pneumoniae* strains.

Strains	Initial MIC (μg/ml)	MIC (μg/ml) after treatment with different concentrations of amikacin			Proportion of resistant subpopulation after treatment with different concentrations of amikacin			MIC (μg/ml) after subculture for 7days without amikacin			Proportion of resistant subpopulation after subculture for 7days without amikacin		
		2× MIC	4× MIC	8× MIC	2× MIC (%)	4× MIC (%)	8× MIC (%)	2× MIC	4× MIC	8× MIC	2× MIC (%)	4× MIC (%)	8× MIC (%)
S38	32 (I^a^)	32 (I)	64 (R^b^)	128 (R)	27.32	72.84	97.34	32 (I)	64 (R)	128 (R)	23.25	60.97	95.89
S33	4 (S^c^)	8 (S)	32 (I)	64 (R)	0.0014	30.15	79.8	4 (S)	8 (S)	32 (I)	0.0012	0.0129	20.94
S30	4 (S)	8 (S)	16 (S)	32 (I)	0.0237	0.416	20.83	4 (S)	8 (S)	16 (S)	0.0194	0.174	0.199
S28	4 (S)	8 (S)	8 (S)	64 (R)	0.0029	0.277	76.92	4 (S)	4 (S)	32 (I)	0.0011	0.0046	13.86
S9	2 (S)	4 (S)	16 (S)	64 (R)	0.0076	0.196	85.53	4 (S)	8 (S)	32 (I)	0.0075	0.0183	21.3
F48	2 (S)	4 (S)	16 (S)	32 (I)	0.0214	0.99	15.09	4 (S)	8 (S)	16 (S)	0.0031	0.012	0.184
F50	2 (S)	4 (S)	8 (S)	32 (I)	0.0224	0.043	10.98	2 (S)	4 (S)	16 (S)	0.0014	0.0024	0.095
F35	8 (S)	8 (S)	64 (R)	256 (R)	0.0673	86.79	98.76	8 (S)	64 (R)	128 (R)	0.0679	81.84	97.87
H28	4 (S)	8 (S)	16 (S)	32 (I)	0.0079	0.48	33.5	4 (S)	8 (S)	16 (S)	0.0001	0.0007	0.029
H628	16 (S)	32 (I)	64 (R)	128 (R)	50.62	74.81	99.8	8 (S)	16 (S)	32 (I)	0.0023	0.0079	10.72
17-3	2 (S)	4 (S)	8 (S)	32 (I)	0.0024	0.031	12.19	4 (S)	4 (S)	16 (S)	0.0016	0.0012	0.019
17-4	8 (S)	16 (S)	32 (I)	64 (R)	0.0086	12.18	87.8	4 (S)	16 (S)	32 (I)	0.0011	0.0096	9.34
17-10	8 (S)	32 (I)	64 (R)	128 (R)	73.68	84.4	92.8	8 (S)	16 (S)	32 (I)	0.0019	0.247	19.35

a *I: K. pneumoniae strains were intermediately resistant to antibiotics*.

b *R: K. pneumoniae strains were resistant to antibiotics*.

c *S: K. pneumoniae strains were susceptible to antibiotics*.

### MLST Analysis of the Amikacin-Heteroresistant *K. pneumoniae*

In this experiment, a total of five STs were found in the amikacin-heteroresistant *K. pneumoniae* strains. These STs were same as the parental strains. ST11 (8/13) was a predominant clone type in these strains. ST2637 (2/13), ST2324 (1/13), ST307 (1/13), and ST761 (1/13) were also identified, and these STs did not belong to CC11. Compared to ST11, all seven housekeeping genes showed loci variations in ST2637 and ST761. ST307 (*phoE* and *gapA*) and ST2324 (*mdh* and *tonB*) have only two housekeeping genes which are identical to ST11.

### Growth Curves of Amikacin-Resistant Parental *K. pneumoniae* and Their Resistant Subpopulation

**Figure 2:**
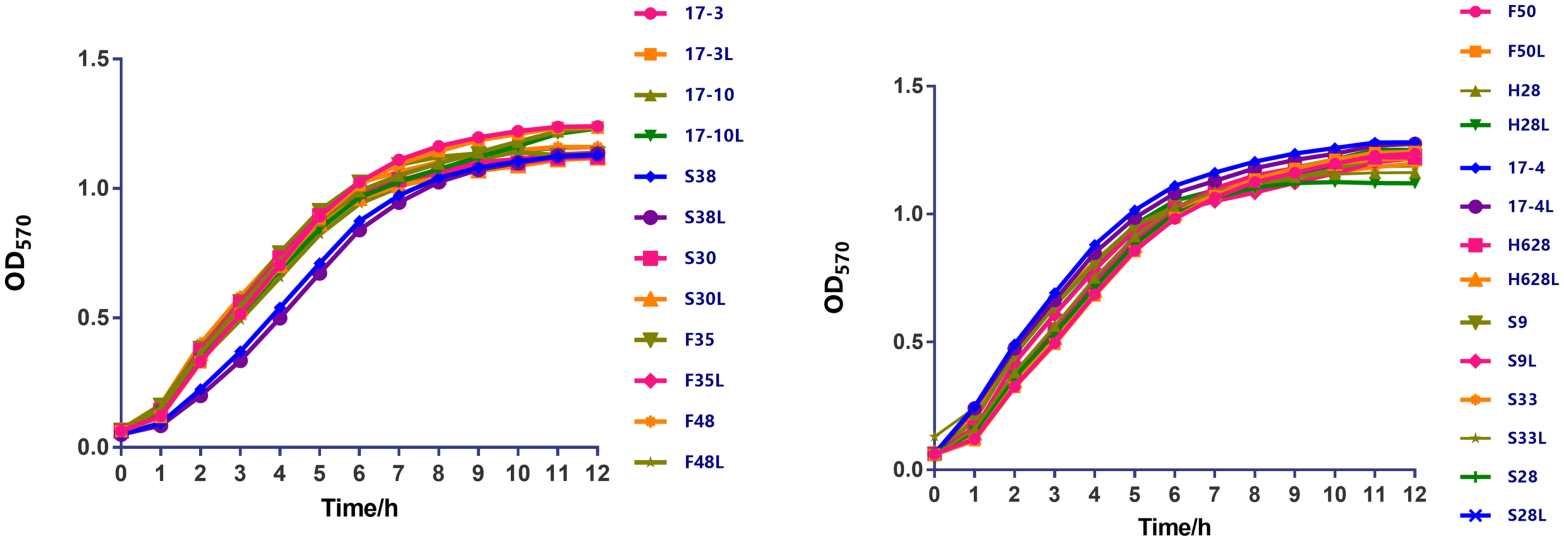
The growth kinetics of amikacin-heteroresistant *K. pneumoniae* isolates and the resistant subpopulation were determined in the absence of selective pressure. During a 12-h period, no significant difference was observed between those strains.

Growth curves among amikacin-resistant *K. pneumoniae* strains and their resistant subpopulation revealed that all the tested strains displayed no difference in growth rate (Figure 2, in the current study, resistant subpopulation was named by adding an L suffix to the name of the parental strain).

### Results of Biofilm Formation

**Figure 3:**
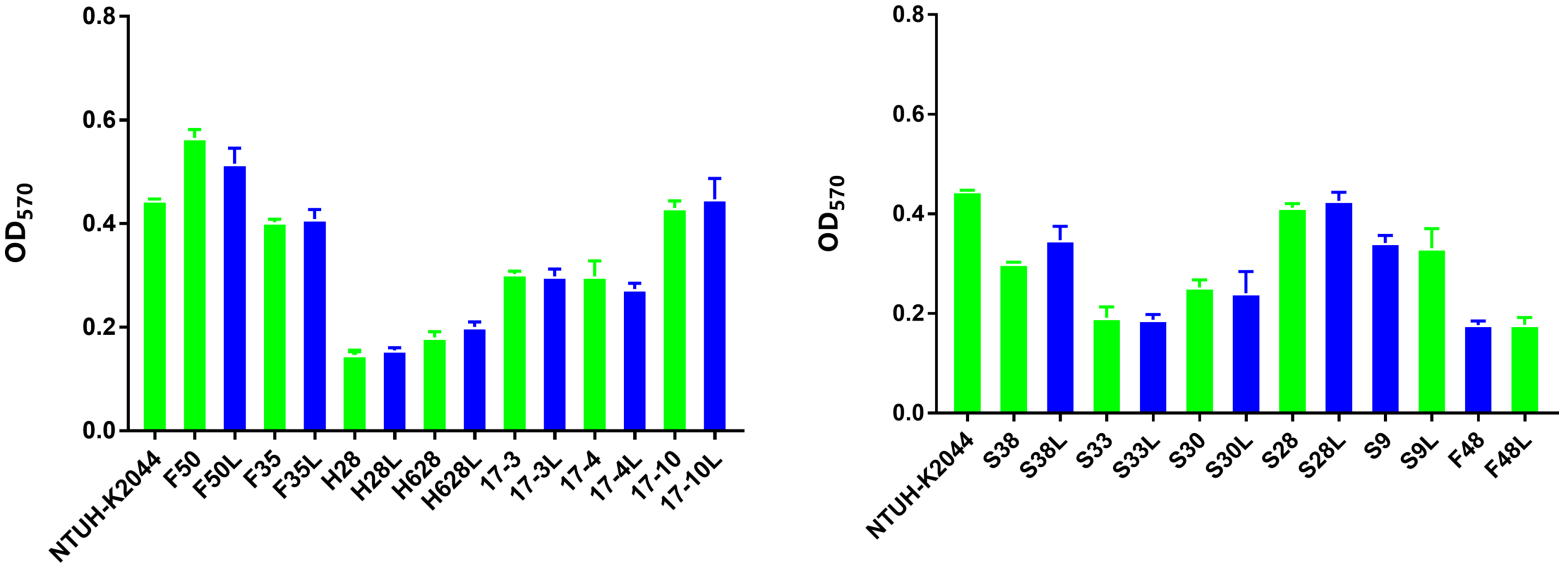
Biofilm formation of the amikacin-heteroresistant *K. pneumoniae* isolates and the resistant subpopulation. Each assay was performed in triplicate and repeated four times. The ability to form biofilms (OD_570_) corresponds to mean values of three replicates for each strain.

The mean OD values obtained by the biofilm formation analysis are plotted in Figure 3. On the basis of the ODc cutoff=0.147, 42.31% (11/26) of the tested isolates were classified as weak biofilm producers (mean ODs=0.198±0.237), 57.69% (15/26) as moderate biofilm producers (mean ODs=0.388±0.079). However, there was no difference in biofilm formation among the amikacin-resistant parental strain and the resistant subpopulation.

### Results of the Expression of Aminoglycoside Resistance Genes, Porin Genes, and 16sRNA Methylase Genes

**Figure 4:**
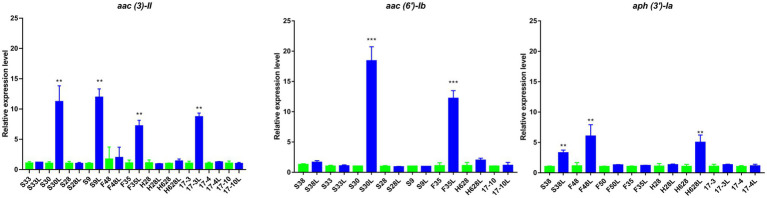
Results of the expression of aminoglycoside resistance genes in amikacin-heteroresistant strains and the resistant subpopulation. The expression levels were detected by qRT-PCR, ^**^statistically significant (*p*<0.01), and ^***^statistically significant (*p*<0.001).

Quantitative RT-PCR analysis revealed that amikacin-resistant subpopulation S30L and F35L had increased expression of *aac (6′)-Ib* compared to the amikacin-resistant parental strains S30 and F35 (18.22- and 11.4-fold increased expression, respectively), which was statistically significant (*p*<0.05). Amikacin-resistant subpopulation S38L, F48L, and H628L had increased expression of *aph (3′)-Ia* compared to their amikacin-resistant parental strains (3.27-, 5.39-, and 4.83-fold increased expression, respectively), which was statistically significant (*p*<0.05). Amikacin-resistant subpopulation S30L, S9L, F35L, and 17-3L had increased expression of *aac (3)-II* compared to their amikacin-resistant parental strains (10.96-, 11.85-, 6.73-, and 8.41-fold increased expression, respectively) which was statistically significant (*p*<0.05; Figure 4). Moreover, no differences were observed in the expression of *ompK35* and *ompK36* among the tested isolates (*p*>0.05; Supplementary Figure S1).

### Whole-Genome Sequences, General Genomic Features, Single-Nucleotide Variants, and Amino Acid Alteration Analysis

In this study, the complete genomic sequences of S38 and S38L were determined. The general genomic features are summarized in Supplementary Table S3 in the supplementary material. Different nucleotide and resultant amino acid alterations were also identified (Table 5). In summary, the nucleotide and resultant amino acid variations were identified in four sites in *parB* gene (Ala 226 Val, Ile 236 Thr, Gln 476 His, and Ser 563 Phe), in one site in the gene encoding carbohydrate porin (Ile 82 leu), in two sites in the gene encoding single-stranded DNA-binding protein (Trp 16 Arg and His 56 Arg), in five sites in the gene encoding threonine dehydrogenase (Met 30 Ile, Ser 33 Cys, Ser 42 Cys, Val 56 Glu, and Arg 93 Trp), in four sites in the gene encoding hypothetical protein (Arg 16 Ser, Ala 54 Glu, Val 62 Leu, and Val 75 Ile), and in three sites in the gene encoding DUF905 domain-containing protein (Val 47 Ile, Phe 74 Tyr, and Glu 77 Gly). As shown, amino acid variations in one site in the gene encoding sequence-specific DNA-binding transcription factor (Ile 134 Met) and in one site in the gene encoding capsid size determination protein (Glu 47 Ala) were also identified.

**Table 5:** Nucleotide and amino acid substitutions in amikacin-heteroresistant *K. pneumoniae* S38 and the resistant subpopulation S38L.

Isolates	Gene or CDs^a^/Product or function	Mutations	
		Nucleotide	Amino acid
S38/S38L	*parB*, accurate chromosome segregation in bacteria	C 678 T T 708 C G 1248 T C 1688 T	Ala 226 Val Ile 236 Thr Gln 476 His Ser 563 Phe
S38/S38L	CDs, Capsid size determination protein Sid	A 140 C G 141 A	Glu 47 Ala
S38/S38L	CDs, Carbohydrate porin	A 244 C C 246 G	Ile 82 leu
S38/S38L	CDs, Sequence-specific DNA-binding transcription factor	A 402 G	Ile 134 Met
S38/S38L	Ssb, Single-stranded DNA-binding protein	T 46 A A 167 G	Trp 16 Arg His 56 Arg
S38/S38L	CDs, Threonine dehydrogenase	G 90 T A 97 T A 124 T T 167 A C 277 T	Met 30 Ile Ser 33 Cys Ser 42Cys Val 56 Glu Arg 93 Trp
S38/S38L	CDs, Hypothetical protein PUUH_pUUH2392p0081	C 46 A C 161 A G 184 C G 223 A	Arg 16 Ser Ala 54 Glu Val 62 Leu Val 75 Ile
S38/S38L	CDs, DUF905 domain-containing protein	G 139 A T 221 A A 230 G	Val 47 Ile Phe 74 Tyr Glu 77 Gly

a *CDs, complete coding sequence*.

## Discussion

In this study, the prevalence of the clinical amikacin-heteroresistant *K. pneumoniae* strains was low, the majority of the heterogeneous phenotypes (84.61%) was unstable, and the MIC fully or partially reverted back to the level of susceptibility of the parental isolates. Recent studies showed that slow growth was linked to the emergence of colistin heteroresistance in *A. baumannii* and biofilm formation was related to colistin heteroresistance in *K. pneumoniae* (Silva et al., 2016; Rodriguez et al., 2019). However, there were no changes in the growth curves and the ability to produce biofilm among the tested strains in this study. The results showed that slow growth and biofilm formation are not the underlying mechanisms for amikacin heteroresistance. MLST analysis of the amikacin-heteroresistant *K. pneumoniae* isolates was performed and the ST11 was a predominant clone type in these strains. As previously reported, ST11 was the most predominant epidemic type in Mainland China. If not effectively controlled, the ST11 clone could spread widely in the future (Tian et al., 2019). In recent years, studies have shown that the decreased expression of the porin gene *ompC* may be related to kanamycin heteroresistance in *Salmonella enterica* and the amplification of aminoglycoside resistance gene *aadBc* is the underlying mechanisms for tobramycin heteroresistance in *A. baumannii* (Sánchez-Romero and Casadesús, 2014; Anderson et al., 2018). Unlike the data obtained from previous reports, no difference was observed in the expression of porin genes *ompK35* and *ompK36* in the tested isolates. The results indicated that porin may not be associated with amikacin heteroresistance in *K. pneumoniae* strains. It is also found that some of the amikacin-heteroresistant *K. pneumoniae* isolates do not have carbapenemases but are resistant to imipenem and meropenem. Because porin is not linked to aminoglycoside resistance, we speculate that the reduced susceptibility to imipenem or meropenem might be due to porins, which is already established. It is worth noting that some resistant subpopulation had increased expression of aminoglycoside resistance genes *aac (6′)-Ib*, *aph (3′)-Ia*, and *aac (3)-II* compared with their amikacin-resistant parental strains. These results indicated that the increased expression of aminoglycoside resistance genes detected in the amikacin-heteroresistant isolates may lead to the appearance of the phenomenon of amikacin heteroresistance. Beyond that, increasing studies have revealed that point mutations, IS insertions, or small deletions in genes associated with antibiotic resistance or pathways result in a small colony variant phenotype and aminoglycoside resistance, such as *ubiJ* and *cydA*, were also linked to the formation of the aminoglycoside heteroresistance in *E. coli*, *K. pneumoniae*, and *Salmonella typhimurium* (Andersson et al., 2019; Nicoloff et al., 2019). However, there were no mutations identified in these genes. On the contrary, several mutations in the genes encoding carbohydrate porin, threonine dehydrogenase, hypothetical protein, single-stranded DNA-binding protein, and *parB* gene were identified. These genes have a variety of roles in bacterial physiological processes, such as carbohydrate uptake and chromosome segregation. We speculated that SNVs identified in this study might be associated with amikacin heteroresistance. However, further studies are still needed to investigate the mechanisms of amikacin heteroresistance and confirm that SNVs are associated with amikacin heteroresistance.

Previous study has shown that heteroresistance could be due to several co-existing subpopulation and mutants isolated from the same subpopulation frequently displayed different MIC levels and stability of the resistant phenotypes (Nicoloff et al., 2019). The results were consistent with what has been observed in this research. Several co-existing subpopulations with different susceptibility to amikacin in amikacin-heteroresistant *K. pneumoniae* strains were observed. This suggested that the analysis of isolated clones, as routinely performed in clinical laboratories, might not always adequately reflect the characteristics of all bacteria present in a clinical sample. Besides, as reported previously, a majority of heteroresistance cases were unstable (Nicoloff et al., 2019). A similar result was obtained in this study that is the majority of the amikacin heterogeneous phenotypes was unstable among a total of 155 strains of *K. pneumoniae*. This type of heteroresistance is difficult to detect because of its intrinsic instability, it is also frequently lost when the antibiotic pressure drops. Because this sort of heteroresistance is difficult to diagnose with standard tests, misdiagnosis of strains with resistant subpopulation as fully susceptible is likely common, with potentially severe consequences in the form of treatment failure. Moreover, recent studies have shown that the resistant subpopulation could be enriched and might cause treatment failure during antibiotic exposure (Andersson et al., 2019). These results were also consistent with what has been observed in this study. The MICs of the amikacin-heteroresistant *K. pneumoniae* strains were increased after exposed to amikacin. This indicated the presence of a switch of heteroresistance to homogeneous high-level resistance during the period of antibiotic treatment.

In general, to the best of our knowledge, this is the first report about the prevalence of amikacin heteroresistance in *K. pneumoniae* strains in China. This research could help us understand the characteristic of amikacin heteroresistance, the switch of heteroresistance to homogeneous high-level resistance, and raise concerns for the emergence of amikacin-heteroresistant *K. pneumoniae* strains. In addition, results obtained in this study indicated that SNVs and the increased expression of aminoglycoside resistance genes detected in amikacin-heteroresistant *K. pneumoniae* strains might be associated with amikacin heteroresistance. However, further studies are still needed to investigate the mechanisms of amikacin heteroresistance.

## Data Availability Statement

The data presented in the study are deposited in the National Center for Biotechnology Information (NCBI), accession numbers JAGFBX000000000 and JAGFBW000000000.

## Author Contributions

YZ and FZ designed the experiments. FZ, JB, and QL performed the experiments. FZ, MD, and XY analyzed the data and wrote the manuscript. All authors contributed to data analysis, drafting or revising the article, gave final approval of the version to be published, and agree to be accountable for all aspects of the work.

## Funding

This research was funded by the grant from the Sichuan Province Science and Technology Project (2020YJ0338), the Major Program of National Natural Science Foundation of China (819931534), and the National Natural Science Foundation of China (31500114).

## Conflict of Interest

The authors declare that the research was conducted in the absence of any commercial or financial relationships that could be construed as a potential conflict of interest.

## Publisher’s Note

All claims expressed in this article are solely those of the authors and do not necessarily represent those of their affiliated organizations, or those of the publisher, the editors and the reviewers. Any product that may be evaluated in this article, or claim that may be made by its manufacturer, is not guaranteed or endorsed by the publisher.

## Footnotes

^1^ http://bigsdb.pasteur.fr/klebsiella/primers_used.html

^2^ http://bigsdb.pasteur.fr/klebsiella/klebsiella.html

^3^ http://topaz.gatech.edu/

^4^ http://rast.nmpdr.org/rast.cgi

^5^ http://www.repeatmasker.org/

^6^ http://www.genome.jp/kegg/

^7^ https://cge.cbs.dtu.dk/services/ResFinder/
