# Supplementary material for: Heteroresistance to Amikacin in Carbapenem-Resistant Klebsiella pneumoniae Strains
Source: Front Microbiol. 2021 Dec 2;12:682239. doi: 10.3389/fmicb.2021.682239 (PMC8753984; doi:10.3389/fmicb.2021.682239)
Supplement: Supplementary file 1 [file Table_1.docx]

Supplementary Material

Table S1. The primers used for quantitative reverse-transcription PCR

| Primer | Sequence (5′–3′) | Gene | Product size (bp) |
| --- | --- | --- | --- |
| OmpK36-F | GCCTGAACTTTGCTCTGC | *ompK36* | 101 |
| OmpK36-R | GCCGTCACCGTTCTGTTT |  |  |
| OmpK35-F | TACGGTCAGTGGGAATACAA | *ompK35* | 102 |
| OmpK35-R | ACCATACTCGCCCGCTTT |  |  |
| AAC (3)-II-F | TCTTCCAAGCATCGGCATCT | *aac (3)-II* | 184 |
| AAC (3)-II-R | GCCTCACGAACTCGGTCA |  |  |
| AAC (6)- IB-F | GTTTCTTCTTCCCACCATCC | *aac (6')-Ib* | 103 |
| AAC (6)- IB-R | AGTCCGTCACTCCATACATTG |  |  |
| APH (3)-IA-F | CCGTCAGCCAGTTTAGTC | *aph (3')-Ia* | 136 |
| APH (3)-IA-R | AATGTCGGGCAATCAGGT |  |  |
| 16S rRNA-F | CGGTGAATACGTTCYCGG | *16S rRNA* | 128 |
| 16S rRNA-R | GGWTACCTTGTTACGACTT |  |  |

Table S2. General information of the 155 strains of *K. pneumoniae* isolated from the hospitalized patients.

| Specimens | Number of isolates (%) | Number of antibiotics heteroresistance (%) | | | |
| --- | --- | --- | --- | --- | --- |
|  |  | Imipenem | Meropenem | Amikacin | Cefoperazone |
| sputum | 107 (69.03 %) | 68 (90.67 %) | 35 (92.11 %) | 13 (100 %) | 20 (83.33 %) |
| blood | 9 (5.81 %) | 0 | 0 | 0 | 1 (4.17 %) |
| urine | 13 (8.39 %) | 7 (9.33 %) | 2 (5.26 %) | 0 | 0 |
| wound secretion | 26 (16.77 %) | 0 | 1 (2.63 %) | 0 | 3 (12.5 %) |

Table S3. General genomic features of the S38 and S38L.

| Feature ^a^ | S38^b^ | S38L^c^ |
| --- | --- | --- |
| Genome size (bp) | 5,717,705 bp | 5,764,723 bp |
| GC content (%) | 58.52 | 58.54 |
| CDs | 5,643 | 5,679 |
| Gene average length (bp) | 892 | 896 |
| Number of tRNAs | 76 | 74 |
| Number of rRNAs | 13 | 12 |
| Number of sRNAs | 49 | 49 |
| Number of genomic islands | 17 | 13 |
| Genes assigned to COGs | 4,506 | 4535 |
| Genes assigned to KEGG | 5,485 | 5516 |
| Genes assigned to PHI | 494 | 503 |
| Genes assigned to VFDB | 399 | 409 |
| Genes related to antibiotic resistence | 19 | 19 |
| Repeated regions (%) | 0.5818 | 0.606 |
| Phage regions | 15 | 11 |
| CRISPRs | 12 | 14 |

^a^ CDs: Complete coding sequence. COGs, Cluster of Orthologous Groups of protein; KEGG, Kyoto Encyclopedia of Genes and Genomes; PHI, Pathogen-Host Interactions database; VFDB, Virulence Factors of Pathogenic Bacteria.

^b^ The accession number of amikacin-heteroresistant *K. pneumoniae* S38 is JAGFBW000000000.

^c^ The accession number of amikacin resistant subpopulation S38L is JAGFBX000000000.


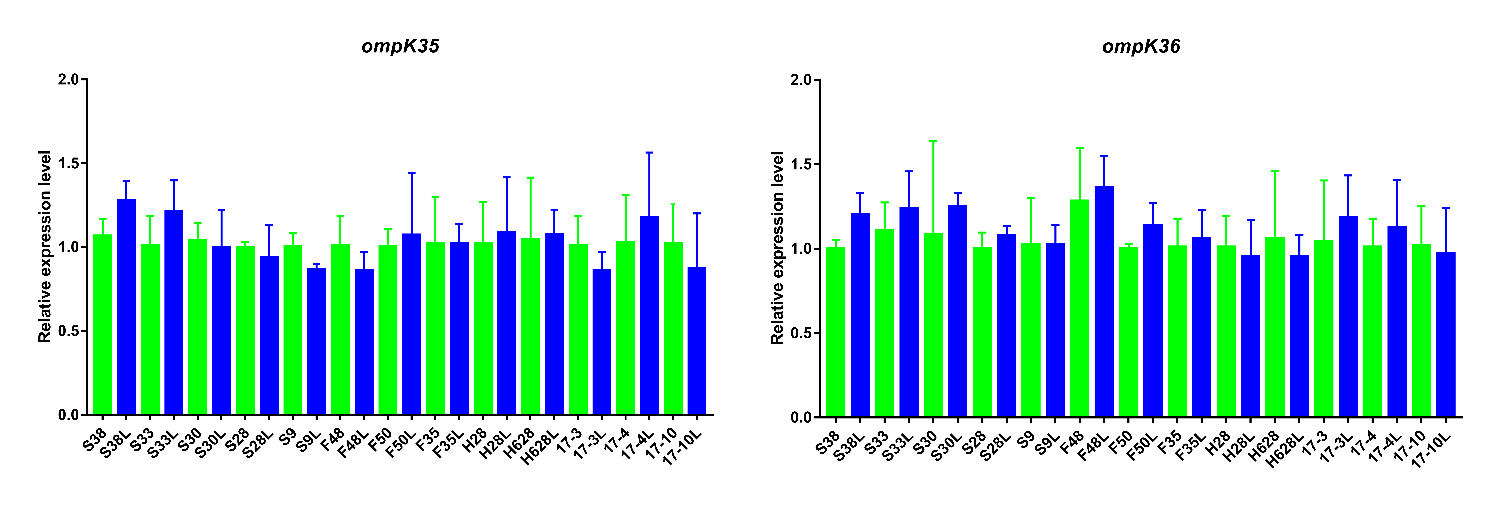
Figure S1.

Figure S1. Results of the expression of porin genes *ompK35* and *ompK36* in amikacin-heteroresistant strains and their resistant subpopulations. The expression levels were detected by qRT-PCR.
